# Transferable conditional brain charts link cortico-subcortical structure to Alzheimer's disease progression

**Published:** 2026-10-15

**Authors:** Nitin Sharma, Sicheng Dai, Francesco A. Mallus, Atharva Kand, Marc Lemnitzer, Minh-Hieu Nguyen, Mebin Muhammed Hamzakoya Sulaikha, Jijia Xing, Mariam Zabihi, Gülce Lale, Gokul Perumbayil Vijayakrishnan, Elias Schreiner, Madhumitha Sivakumar Punitha, Paul Buttkus, Çağatay Yıldız, Poornima Kumar, Thomas Grote, Esten Leonardsen, Francesco Sammarco, Vinod Kumar Jangir, Sarah Sobotta, Barbora Rehák Bučková, Augustijn A. A. de Boer, Konstantinos Tsilimparis, Christian Gaser, Charlotte Fraza, Saige Rutherford, Seyed Mostafa Kia, Andre F. Marquand, Yanwu Yang, Richard Dinga, Thomas Wolfers

## Abstract

Normative models chart how brain measures vary across the lifespan, expressing each individual as a deviation from a healthy reference. However, most regional models of cortical thickness disregard dependencies between cortical and subcortical anatomy, and transferring them to a new site typically requires retraining or a recalibration of location and scale alone. We condition lifespan norms of cortical thickness on subcortical volume, such that each deviation indicates whether an individual's cortex lies within the healthy range given their subcortical anatomy, and make them transferable. In 21,351 healthy individuals from 216 sites, conditioning on ten subcortical structures improved out-of-sample prediction on average, most for the amygdala and hippocampus. Refitting only the intercepts of all four distributional parameters on about 20 healthy individuals, as implemented in our open-source package NormGAMLSS, calibrated most of six unseen sites without retraining, for base and conditional models. In the Alzheimer's Disease Neuroimaging Initiative, global deviation and structural dissociation (the reduction in mean absolute deviation under conditioning) predicted conversion to mild cognitive impairment and dementia beyond the Clinical Dementia Rating and dissociation added beyond global deviation for dementia, even at similar clinically recorded impairment. Conditional norms thus provide relevant, individual-level information.

